# Circulating apelin and chemerin levels in patients with polycystic ovary syndrome: A meta-analysis

**DOI:** 10.3389/fendo.2022.1076951

**Published:** 2023-01-11

**Authors:** Yiming Gao, Caihong Xin, Huaying Fan, Xin Sun, Hongli Wang

**Affiliations:** ^1^ The First Clinical College of China Medical University, Shenyang, China; ^2^ Department of Endocrinology and Metabolism, The Fourth People’s Hospital of Shenyang, Shenyang, China; ^3^ Department of Endocrinology and Metabolism, The First Affiliated Hospital of Soochow University, Suzhou, China; ^4^ Department of Cardiology, The Second Affiliated Hospital of Dalian Medical University, Dalian, China

**Keywords:** apelin, chemerin, polycystic ovary syndrome, PCOS, meta- analysis

## Abstract

**Background:**

Polycystic ovary syndrome (PCOS) is one of the most common gynecological endocrine disorders. Apelin and chemerin are newly identified adipokines, which are higher in obesity and diabetes. Studies have found that the serum apelin and chemerin levels in patients with PCOS are significantly increased. However, other studies showed the opposite results. Therefore, the relationship between those two adipokines and PCOS is still controversial.

**Aim:**

This meta-analysis was conducted to statistically evaluate the apelin and chemerin levels of patients with PCOS.

**Methods:**

We searched the Web of Science, Embase, PubMed, and Google Scholar databases for potential studies. “Polycystic ovary syndrome” or “PCOS” in combination with the terms “apelin” or “chemerin” were used as keywords search titles or abstracts. The publication period examined was between 1990 and 2021. Standardized mean differences (SMD) with corresponding 95% confidence intervals (CIs) were determined as the results of the meta-analysis.

**Results:**

A total of 148 articles were initially retrieved, and 18 qualified articles were finally obtained through preliminary screening and quality evaluation. The publications together contain 1,265 cases and 894 controls. The results of the meta-analysis showed that the circulating chemerin levels in patients with PCOS were significantly higher than those in the controls (SMD: 0.79, 95% CI [0.36, 1.23]), and there was no significant difference in circulating apelin between patients with PCOS and controls (SMD: 0.57, 95% CI [-0.21, 1.35]).

**Conclusions:**

This meta-analysis is the first to evaluate circulating apelin and chemerin levels in patients with PCOS. Our findings suggest that circulating chemerin levels of patients with PCOS are significantly higher than those of healthy controls.

**Systematic review registration:**

https://www.crd.york.ac.uk/PROSPERO/display_record.php?RecordID=218316, identifier CRD42020218316.

## Introduction

Polycystic ovary syndrome (PCOS) is one of the most common gynecological endocrine disorders with a complex pathogenesis (1). It is characterized by dysmenorrhea, amenorrhea, infertility, hairiness, and obesity, all of which seriously affect the physical and mental health of women of childbearing age. PCOS also increases the risk of endometrial cancer, gestational diabetes mellitus, and hyperlipidemia. Approximately 5%–10% of women of childbearing age are affected by PCOS. Patients with PCOS account for 15%–20% of infertility cases (2, 3). Studies have shown that abnormal follicular development in patients with PCOS is not only regulated by sex hormones but is also closely related to disorders in the follicular development microenvironment caused by ovarian autocrine/paracrine dysfunction. Recent studies suggested that insulin resistance may initiate PCOS development (4).

Apelin and chemerin are newly identified adipokines. Apelin is an APJ receptor ligand, is widely expressed in different organs, and plays an important role in glucose and lipid metabolism (5). Recent studies found that serum apelin levels are significantly correlated with type 2 diabetes mellitus and obesity (6, 7). Chemerin, also called TIG2 or RARRES2, is secreted as an 18 kDa precursor protein (chem163s), which can be transformed into a 16 kDa active molecule only after C-terminal cleavage (8). The precursor protein has multiple restriction sites, which can produce a variety of subtypes upon treatment with different proteases. Chemerin also contributes to adipogenesis, glucose homeostasis, food intake, and body weight and is associated with elevated levels of obesity, diabetes, and cancer (9–11).

Studies have found that serum apelin and chemerin levels in patients with PCOS are significantly increased (12–15), and are potential targets for the treatment of PCOS (16, 17). However, other studies showed that the serum apelin and chemerin levels of PCOS patients are lower than those of healthy individuals (18, 19). Therefore, the relationship between these adipokines and PCOS remains controversial. This meta-analysis aimed to statistically evaluate apelin and chemerin levels in patients with PCOS.

## Methods

### Search design

We searched the Web of Science, Embase, PubMed, and Google Scholar databases for potential studies. “Polycystic ovary syndrome” or “PCOS” in combination with the terms “apelin” or “chemerin” were used as keywords search titles or abstracts. The full electronic search strategy is provided in the **Supplementary Data Sheet 1**. The publication period examined was between 1990 and 2021. Concurrently, manual retrieval of relevant literature was performed and the references included in clinical trials were consulted to uncover relevant studies that might have been omitted. This review and meta-analysis were conducted according to the recommendations of the Cochrane Collaboration and following the PRISMA statement. The PRISMA list is provided in the **Supplementary Data Sheet 2** and the PROSPERO registration number is CRD42020218316.

### Inclusion criteria

The studies included in this meta-analysis met the following criteria: (1) case-controlled or prospective design; (2) detailed data on circulating apelin or chemerin levels in patients with PCOS and healthy controls; and (3) written in English. All the patients with PCOS included in the studies had no medical history or evidence of diabetes, hypertension, hyperprolactinemia, thyroid disease, Cushing’s syndrome, and congenital adrenal hyperplasia. Patients taking drugs such as insulin-sensitizing drugs, oral contraceptives, corticosteroids, anti-androgens, and gonadotropin-releasing hormone agonists or antagonists within 3 months were also excluded from the study.

### Data extraction and risk of bias

Two independent evaluators screened the studies according to the inclusion and exclusion criteria. First, the evaluators read the topic and abstract and eliminated duplicate studies and those who did not meet the inclusion criteria. Next, they read the full text of the documents marked for inclusion and cross-checked the results. Finally, the two reviewers discussed and came to consensus on any publications with objections. If they still could not reach an agreement, a third researcher was invited for further evaluation. For documents with questions or missing data, we contacted the author or corresponding author to obtain as much confirmation or supplemental data as possible. The extracted content of the original publication data included the first author, publication year, study period, region, study design, and details of cases and controls.

The Newcastle Ottawa Scale (NOS) was used as the standard to evaluate the quality of the included literature. The NOS is applicable to the evaluation of cohort and case-controlled studies. It consists of three parts: selection of exposure and control populations, comparability, and evaluation of exposure or outcome. It has eight entries. NOS uses the semi-quantitative principle of a star scale to evaluate the quality of literature with a maximum of nine stars (20, 21).

### Statistical analysis

Standardized mean differences (SMD) with corresponding 95% confidence intervals (CIs) were determined as the results of the meta-analysis. Cochran’s Q test and I^2^ statistics were used to test the heterogeneity among the studies. When I^2^ ≤ 50%, there was no heterogeneity and a fixed effects model was used for combined analysis. When I^2^ > 50%, there was significant heterogeneity and the random effects model was used for combined analysis. The stability of the meta-analysis results was evaluated by a sensitivity analysis. Low-quality literature was excluded and the impact of a single study on the overall research results was excluded for each study. Begg’s test was used to analyze publication bias. The significance level was set at *P* < 0.05. Stata 12.0 (College Station, TX, USA) was used for analysis.

## Results

**Figure 1:**
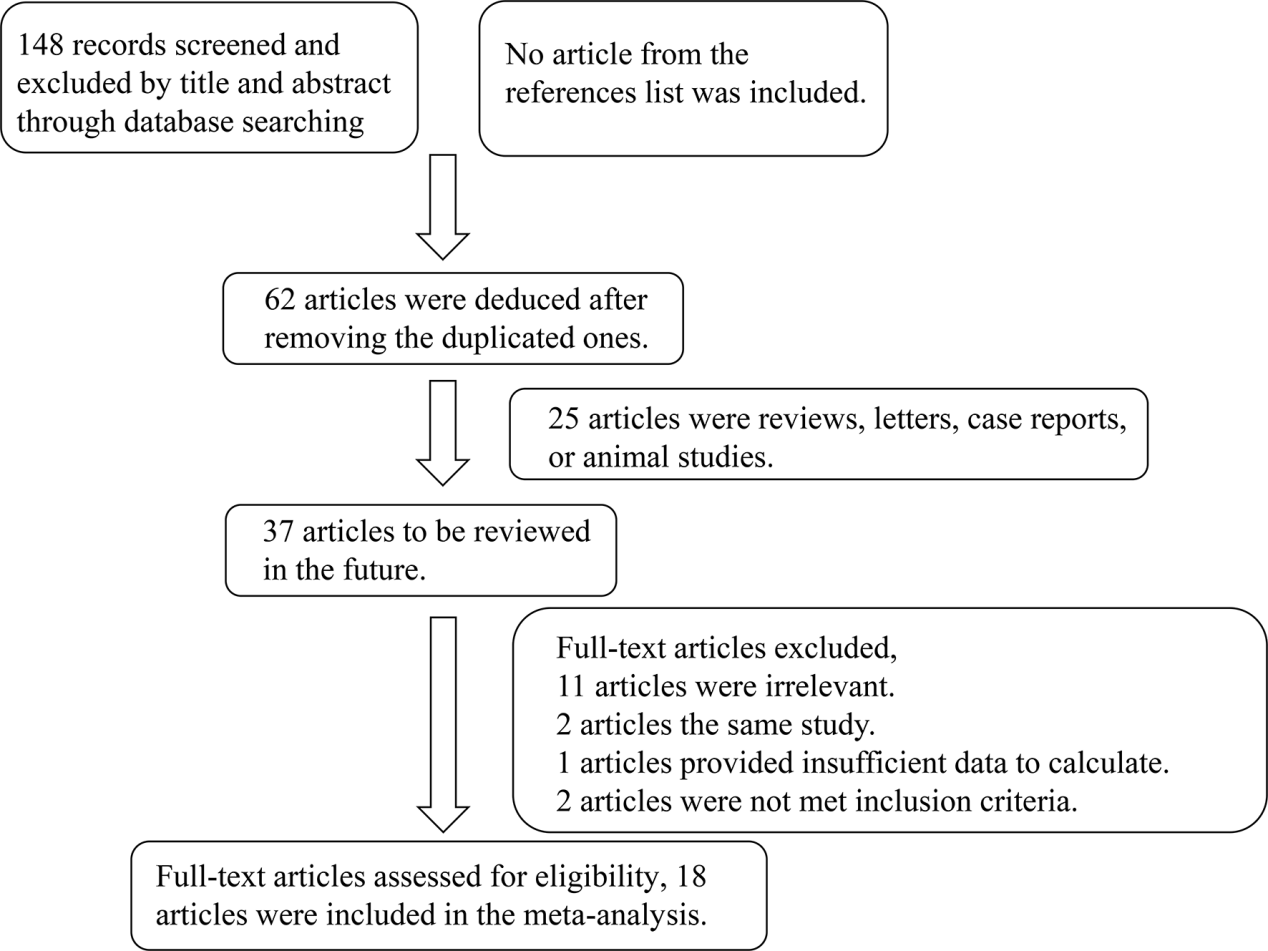
Flowchart of the detailed procedure for the inclusion or exclusion of selected studies.

A total of 148 articles were initially retrieved, and 18 qualified articles were finally obtained through preliminary screening and quality evaluation (5–25). The publications together contain 1,265 cases and 894 controls (12–15, 18, 19, 22–33). The literature retrieval process is shown in **Figure 1** and the baseline data and quality evaluation of the included case-controlled studies are shown in **Table 1**.

**Table 1:** Study characteristics of the published studies included in the meta-analysis.

Author	Publication Year	Study Period	Region	Study design	Case(n)	Control(n)	Case factor	Control factor	Indicator
Goren	2011	–	Turkey	Case-control study	32	31	PCOS patients, 15 – 35 years, BMI 22.51 ± 3.20 kg/m^2^	healthy volunteers, 15 – 35 years, BMI 21.94 ± 1.63 kg/m^2^	Apelin
Chang	2011	–	China	Case-control study	50	34	PCOS patients recruited from the outpatient Department of Obstetrics and Gynecology, 24.8 ± 5.0 years, BMI 22.2 ± 4.1 kg/m^2^	healthy controls, 28.9 ± 5.0 years, BMI 21.0 ± 2.7 kg/m^2^	Apelin
Cekmez	2011	2007 – 2008	Turkey	Case-control study	48	37	Obese PCOS patients recruited among the adolescents who attended the outpatient clinic of the Department of Pediatric Endocrinology, 16.9 ± 0.3 years, BMI 35.1 ± 4.3 kg/m^2^	Obese, healthy children enrolled from patients who attended the hospital for minor illnesses such as the common cold, conjunctivitis etc, 17.2 ± 0.2 years, BMI 30.7 ± 2.2 kg/m^2^	Apelin
Choi	2012	–	Korea	Prospective observational study	82	33	PCOS recruited from outpatients of the Department of Obstetrics and Gynecology, 24.51 ± 5.02 years, BMI 20.27 ± 2.34 kg/m^2^	women visited our hospital for annual comprehensive medical examinations without specific health problems, 24.58 ± 2.72 years, BMI 19.88 ± 1.56 kg/m^2^	Apelin
Wang	2014	July 2012 – April 2013	China	Case-control study	67	20	PCOS patients recruited consecutively from infertility and endocrine clinics, 24.46 ± 4.97 years, BMI 25.77 ± 3.23 kg/m^2^	healthy volunteers, 23.55 ± 4.99 years, BMI 22.49 ± 2.29 kg/m^2^	Chemerin
Ademoglu	2014	–	Turkey	Case-control study	70	38	newly diagnosed or untreated PCOS patients, 25.1 ± 5.7 years, BMI 27.4 ± 7.0 kg/m^2^	aged–match healthy women, 26.2 ± 4.9 years, BMI 21.3 ± 2.7 kg/m^2^	Chemerin
Guzel	2014	2011 – 2012	Turkey	Case-control study	80	57	PCOS patients recruited from the outpatient endocrinology and gynecology clinics, 25.73 ± 6.02 years, BMI 26.23 ± 6.58 kg/m^2^	healthy volunteers, 24.89 ± 4.27 years, BMI 24.54 ± 4.29 kg/m^2^	Chemerin
Benk	2014	–	Turkey	Case-control study	30	30	PCOS patients recruited from the Outpatient Clinic of Obstetrics and Gynaecology Department, 22.46 ± 4.11 years, BMI 20.76 ± 2.08 kg/m^2^	BMI- and age-matched healthy volunteers, 23.66 ± 7.08 years, BMI 20.04 ± 2.22 kg/m^2^	Apelin
Altinkaya	2014	–	Turkey	Case-control study	45	45	PCOS patients, 23.5 ± 5.3, BMI 25.3 ± 3.9 kg/m^2^	age-matched women who had regular menses and no clinical or biochemical hyperandrogenism or PCO were eligible, 25.1 ± 5.7 years, BMI 22.8 ± 2.3 kg/m^2^	Apelin
Yang	2015	January 2013 – June 2014	China	Case-control study	118	114	PCOS patients recruited from the outpatient endocrinology and gynecology clinics, 25.07 ± 4.27 years, BMI 24.63 ± 4.37 kg/m^2^	healthy volunteers with normal ovulatory menstruation, 24.62 ± 3.69 years, BMI 23.08 ± 3.34 kg/m^2^	Chemerin
Huang	2015	March 2012 – June 2014	China	Case-control study	148	88	newly diagnosed PCOS patients, 28.69 ± 5.69 years, BMI 25.80 ± 5.18 kg/m^2^	healthy volunteers, 25.79 ± 5.11 years, BMI 23.24 ± 3.05 kg/m^2^	Chemerin
Olszanecka–Glinianowicz	2015	2010 – 2011	Poland	Prospective observational study	83	67	newly diagnosed PCOS patients, 25.4 ± 5.5 years, BMI 29.4 ± 8.8 kg/m^2^	regularly menstruating women without clinical symptoms of hyperandrogenism, 25.7 ± 4.9 years, BMI 28.3 ± 7.0 kg/m^2^	Apelin
Guvenc	2016	–	Turkey	Case-control study	40	30	PCOS patients recruited from the endocrinology and gynecology, 25.40 ± 5.62 years, BMI 24.87 ± 5.02 kg/m^2^	women who had visited the clinic for non–hormonal or non–menstrual irregularities. 31.50 ± 7.5 years, BMI 23.7 ± 4.46 kg/m^2^	Chemerin
Kiyak Caglayan	2016	–	Turkey	Prospective observational study	55	55	PCOS patients recruited from the obstetrics and gynecology polyclinic, 26.42 ± 4.77 years, BMI 26.81 ± 4.76 kg/m^2^	age- and BMI-matched healthy volunteer, 28.44 ± 6.28 years, BMI 25.78 ± 4.93 kg/m^2^	Apelin
Martinez–Garcia	2019	–	Spain	Case-control study	17	17	newly diagnosed PCOS patients, 26.82 ± 6.87 years, BMI 30.12 ± 7.59 kg/m^2^	age- and BMI-matched healthy volunteers recruited from the hospital’s staff and by noticeboard advertising, 26.47 ± 5.34 years, BMI 29.12 ± 7.33 kg/m^2^	Chemerin
Foda	2019	January 2016 – July 2018	Egypt	Prospective observational study	100	70	untreated PCOS patients recruited from Department of Obstetrics and Gynecology, 21 –26 years, BMI 27.90 ± 3.37 kg/m^2^	women with regular periods and normal findings on pelvic ultrasound scan, 26.47 ± 5.34 years, BMI 27.67 ± 3.90 kg/m^2^	Chemerin
Ozegowska	2019	2014 – 2016	Poland	Case-control study	94	68	PCOS patients recruited at the Department of Infertility and Reproductive Medicine, 27.0 (24.0 – 29.0) years, BMI 21.0 (20.0 – 22.6) kg/m^2^	age- and BMI-matched healthy volunteers with regular menstrual cycles, 28.0 (26.0 – 30.0) years, BMI 20.5 (19.5 – 22.3) kg/m^2^	Apelin
Abruzzese	2020	April 2009 – September 2017	Argentina	Case-control study	106	60	PCOS patients recruited from Department of Endocrinology, 26.42 ± 5.36 years, BMI 31.8 (18.5 – 49) kg/m^2^	unrelated women recruited from voluntary donors at the Department of Hemotherapy and Endocrinology, 27.70 ± 4.85 years, BMI 23 (18 – 45.65) kg/m^2^	Chemerin

### Results of the meta-analysis

**Figure 2:**
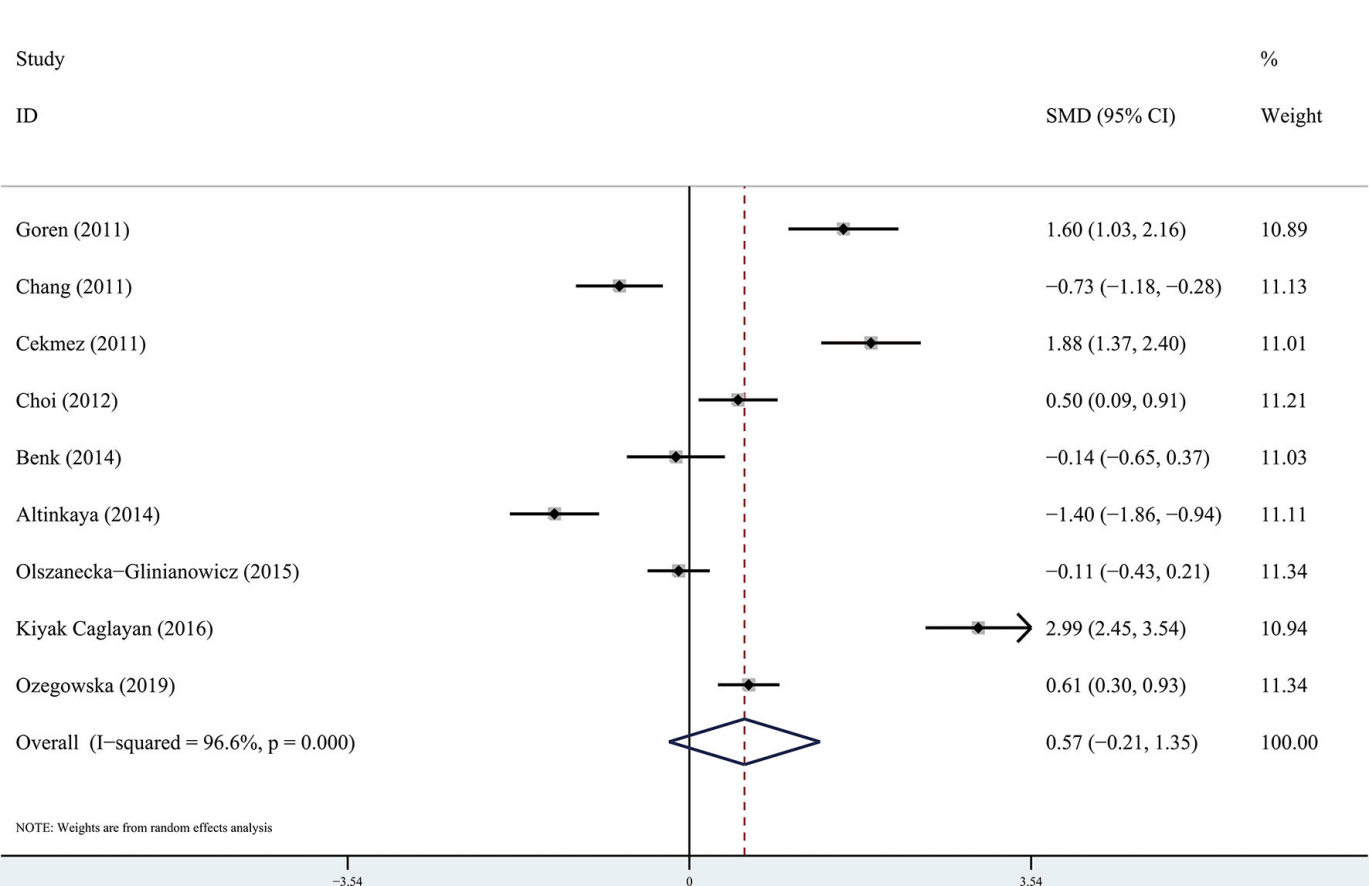
Forest plots of circulating apelin levels in patients with polycystic ovary syndrome compared with controls. Diamond represents the pooled odds ratio at 95% confidence interval.

**Figure 3:**
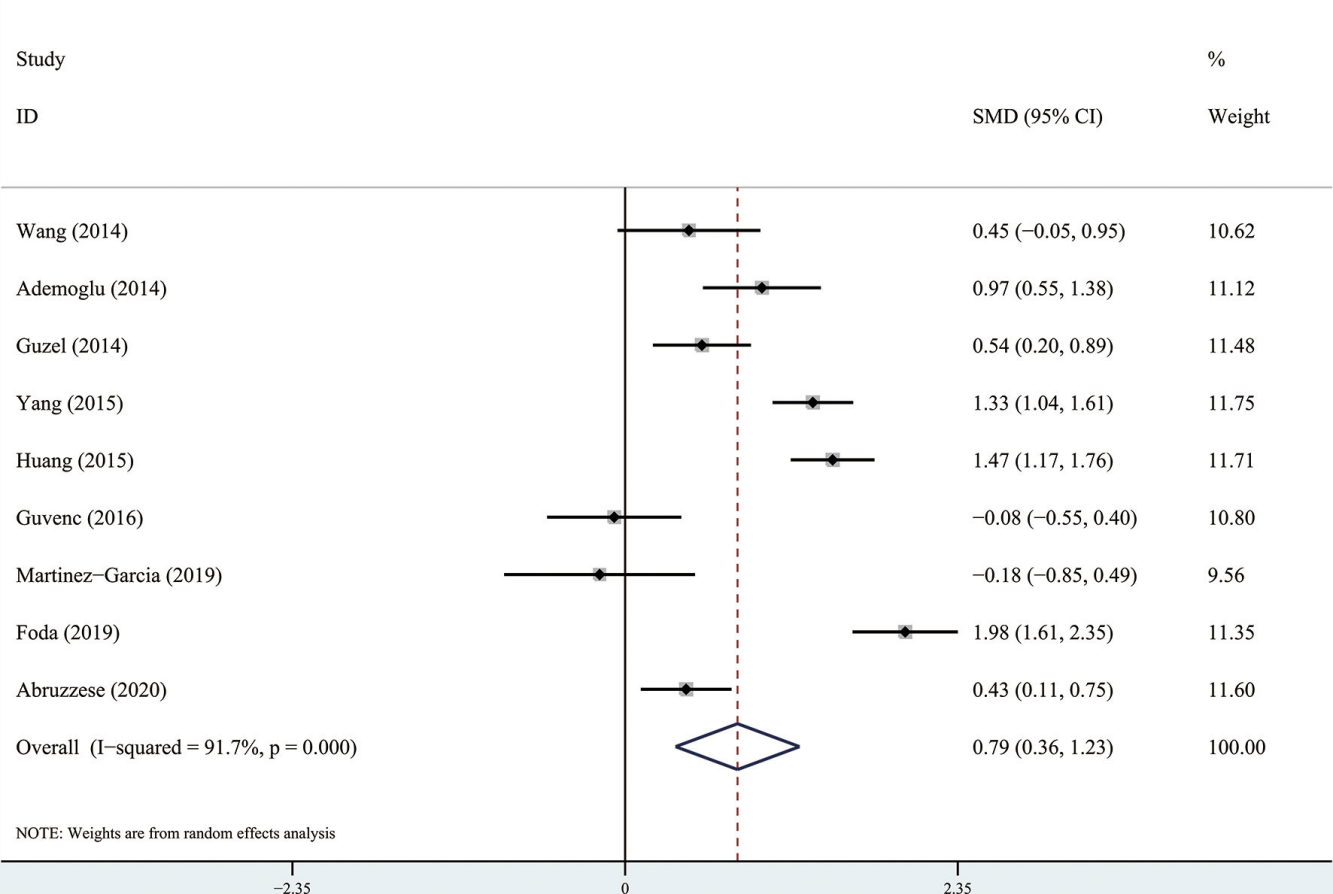
Forest plots of circulating chemerin levels in patients with polycystic ovary syndrome compared with controls. Diamond represents the pooled odds ratio at 95% confidence interval.

The results of the meta-analysis showed that there was no significant difference in circulating apelin between patients with PCOS and controls (SMD: 0.57, 95% CI [-0.21, 1.35]; I^2^ = 96.6%). Forest plots of circulating apelin levels in patients with PCOS compared with controls are shown in **Figure 2**. The circulating chemerin levels in patients with PCOS were significantly higher than those in the controls (SMD: 0.79, 95% CI [0.36, 1.23]; I^2^ = 91.7%). Forest plots of circulating chemerin levels are shown in **Figure 3**. The funnel plots of circulating apelin and chemerin were presented in **Supplementary Figures 1**, **2**.

### Sensitivity analysis and publication bias

**Figure 4:**
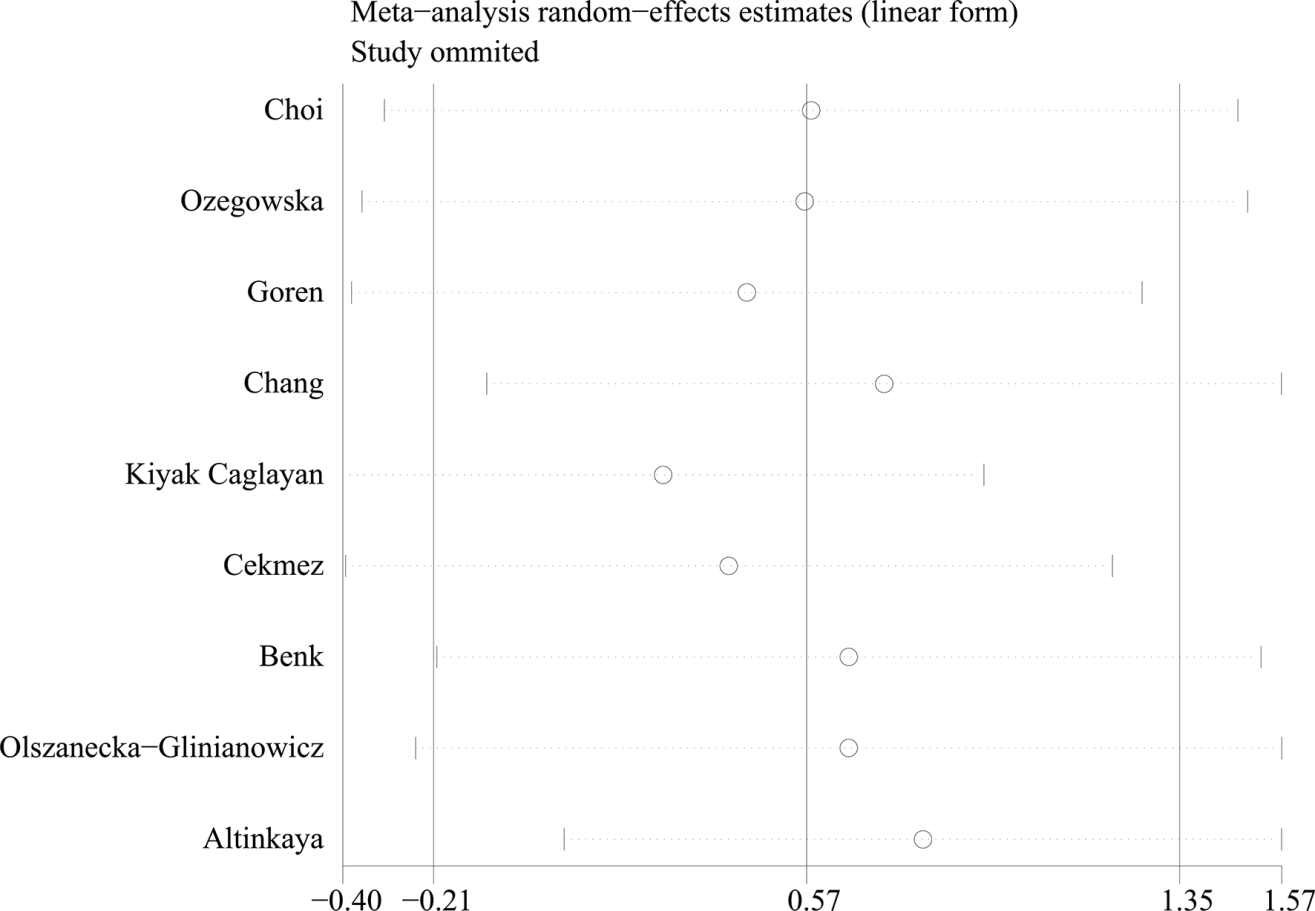
The sensitivity analysis results of circulating apelin levels in patients with polycystic ovary syndrome compared with controls.

**Figure 5:**
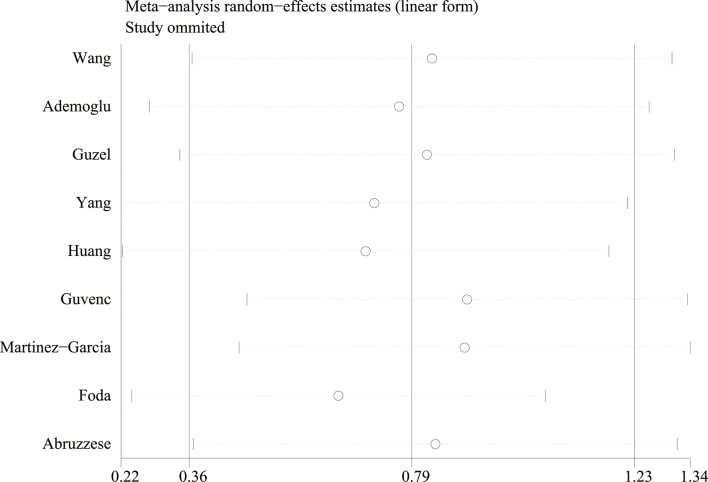
The sensitivity analysis results of circulating chemerin levels in patients with polycystic ovary syndrome compared with controls.

Using the sensitivity analysis by excluding individual studies one by one, the results showed little difference, suggesting that the results of this study were relatively credible (**Figures 4**, **5**). A comprehensive search of articles obtained from the database was performed. Begg’s test was also performed to determine whether there was a potential publication bias in the reviewed literature. The results (*P* > 0.05) suggest that there was no publication bias.

## Discussion

This systematic review is the first to evaluate circulating apelin and chemerin levels in patients with PCOS. Although most studies have shown that circulating apelin and chemerin levels in patients with PCOS are higher than those in healthy controls, some found that they are lower. In this meta-analysis, 18 independent studies were included and analyzed. We concluded that the circulating chemerin levels in patients with PCOS were significantly higher than those in the healthy controls (SMD: 0.79, 95% CI [0.36, 1.23]), whereas there was no significant association between circulating apelin and PCOS (SMD: 0.57, 95% CI [-0.21, 1.35]).

A 5-dihydrotestosterone (DHT)-induced rat model was used to simulate the reproductive and metabolic phenotypes of PCOS. These animal experiments showed that recombinant chemerin inhibits basal estradiol secretion in DHT-induced rat granulosa cells. *In vitro*, chemerin suppressed follicle-stimulating hormone-induced progesterone and estradiol secretion in cultured preantral follicles and granulosa cells (34). Chemokine-like receptor-1 (CMKLR1), an orphan G-protein-coupled receptor, is specifically expressed by monocyte-derived dendritic cells, macrophages, and circulating plasmacytoid dendritic cells. Chemerin is a chemoattractant ligand for CMKLR1. *CMKLR1* gene deletion attenuates the effects of chronic DHT treatment on ovarian function in mouse models of DHT-induced PCOS, likely *via* BMP4 signaling (35). Chemerin also reduces IGF-1-induced steroidogenesis and cell proliferation by decreasing the activation of the IGF-1R signaling pathway in primary human granulosa cells (36). Additionally, metformin treatment has been shown to significantly reduce serum chemerin levels in PCOS patients (32, 37). Chemerin treatment *in vitro* stimulates the process of angiogenesis (38). Therefore, Anusha et al. hypothesized that the increased expression of ovarian chemerin protein in PCOS subjects may cause derangements in ovarian steroidogenesis or angiogenesis that may trigger the development and progression of metabolism related reproductive disorder (39). In addition, murine model of polycystic ovaries when treated with pioglitazone and metformin showed improved insulin resistance and abnormal steroid production by attenuating the ovarian chemerin gene expression (40). These findings suggest that chemerin is a novel negative regulator that may contribute to PCOS pathogenesis. However, further investigation is necessary to understand the effects of chemerin on PCOS.

Although circulating apelin levels are significantly associated with diabetes, in our meta-analysis, there was no significant association between circulating apelin levels and PCOS. Apelin is expressed in granulosa cells, follicles, and follicular fluid and participates in the normal development of follicles, selection of dominant follicles, and proliferation and apoptosis of granulosa cells (41, 42). However, the influence of apelin on PCOS pathogenesis seems to be more complicated, as indicated by controversial data regarding the association between apelin levels, HOMA-IR, and body mass index (BMI) (12, 18, 22, 27). More high-quality studies are needed to better support the association between apelin and PCOS.

This meta-analysis aimed to statistically evaluate circulating apelin and chemerin levels in PCOS patients. However, this study had some limitations. Due to the lack of large sample case-controlled studies, most of the studies included in this meta-analysis were small. Additionally, some studies did not use BMI-matched healthy controls. Different detection methods for apelin and chemerin were used in these studies. All these factors may have affected the results; therefore, the results of this meta-analysis should be interpreted cautiously, as further research is needed.

## Conclusion

This meta-analysis is the first to evaluate circulating apelin and chemerin levels in PCOS patients. Our findings suggest that circulating chemerin levels in PCOS patients are significantly higher than those in healthy controls. More high-quality studies are needed to better support the association between serum apelin levels and PCOS.

## Data availability statement

The original contributions presented in the study are included in the article/**Supplementary Material**. Further inquiries can be directed to the corresponding authors.

## Author contributions

XS designed the study. YG and XS searched databases and collected the data. HF and HW assessed the quality of the study. XS performed the analysis. HF and XS wrote the manuscript. All authors contributed to the article and approved the submitted version.

## Supplementary material

Supplementary Data Sheet 1 The full electronic search strategy.

Supplementary Data Sheet 2 Preferred reporting items for systematic review and meta-analyses (PRISMA) checklist

Supplementary Figure 1 The funnel plots of circulating apelin levels in patients with polycystic ovary syndrome compared with controls.

Supplementary Figure 2 The funnel plots of circulating chemerin levels in patients with polycystic ovary syndrome compared with controls.
