# Supplementary material for: Circulating apelin and chemerin levels in patients with polycystic ovary syndrome: A meta-analysis
Source: Front Endocrinol (Lausanne). 2023 Jan 11;13:1076951. doi: 10.3389/fendo.2022.1076951 (PMC9874085; doi:10.3389/fendo.2022.1076951)
Supplement: Supplementary Data Sheet 1 — The full electronic search strategy. [file DataSheet_1.docx]

The full electronic search strategy

PubMed: ("Polycystic ovary syndrome"[Title/Abstract] OR "PCOS"[Title/Abstract]) AND ("apelin"[Title/Abstract] OR "chemerin"[Title/Abstract]).

Web Of Science: ((AB=(PCOS)) OR AB=(polycystic ovary syndrome)) AND ((AB=(apelin)) OR AB=(chemerin))

Embase: ((‘Polycystic ovary syndrome’:ti,ab OR ‘PCOS’:ti,ab) AND (‘apelin’:ti,ab OR ‘chemerin’:ti,ab).

Google Scholar: (polycystic ovary syndrome OR PCOS) AND (apelin OR chemerin)
