# Supplementary figures and images for: Circulating apelin and chemerin levels in patients with polycystic ovary syndrome: A meta-analysis
Source: Front Endocrinol (Lausanne). 2023 Jan 11;13:1076951. doi: 10.3389/fendo.2022.1076951 (PMC9874085; doi:10.3389/fendo.2022.1076951)

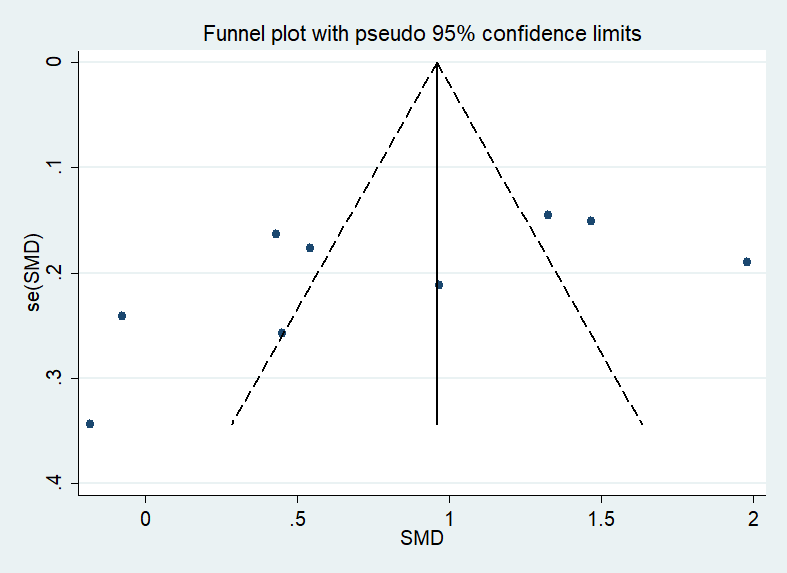

Supplement: Supplementary Figure 1 — The funnel plots of circulating apelin levels in patients with polycystic ovary syndrome compared with controls. [file Image_1.tif]

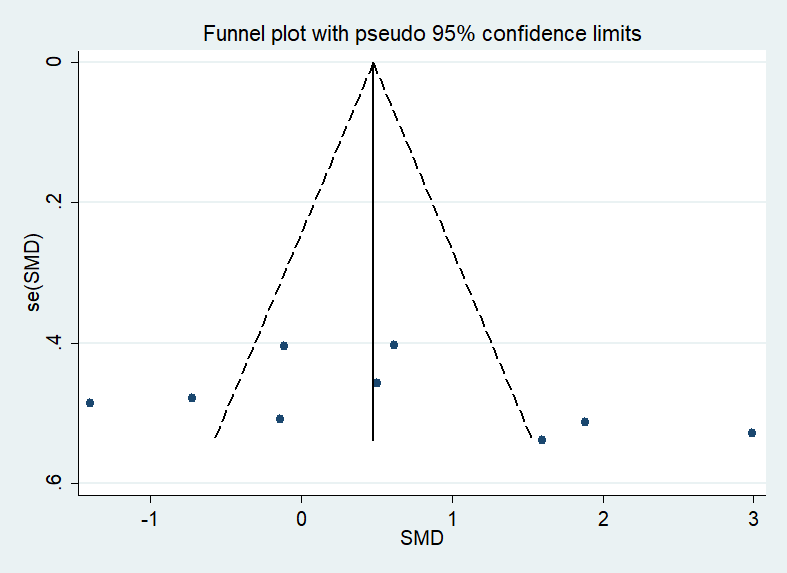

Supplement: Supplementary Figure 2 — The funnel plots of circulating chemerin levels in patients with polycystic ovary syndrome compared with controls. [file Image_2.tif]
